# Circular RNA LONP2 regulates proliferation, invasion, and apoptosis of bladder cancer cells by sponging microRNA-584-5p

**DOI:** 10.1080/21655979.2022.2054753

**Published:** 2022-03-31

**Authors:** Xu Zhang, Hao Xiong, Yong Zhao, Shengqiang Lin, Xiang Huang, Cheng Lin, Shihui Mao, Demin Chen

**Affiliations:** Department of Urology, The Affiliated Nanping First Hospital of Fujian Medical University, Nanping, Fujian, China

**Keywords:** CircLONP2, proliferation, invasion, apoptosis, bladder cancer

## Abstract

Bladder cancer (BC) is the most frequent type of urinary tumor and a barely treatable disease. Although extensive efforts have been invested in the research of BC, the underlying etiology and pathophysiology remain unclear. CircLONP2 is a circular RNA implicated in the development of many cancers, and miR-584-5p and YAP1 have been reported to contribute to the progression of BC. In this research, we presented novel evidence supporting circLONP2/miR-584-5p/YAP1 axis as a novel regulatory module in the progression of BC. We analyzed the expression of circLONP2 between precancerous BC samples and normal tissues using a published RNA-seq dataset. The expression of circLONP2 was also validated in clinical samples and cell lines by quantitative RT-PCR. Small interfering RNA (siRNA) and miRNA inhibitor was utilized to modulate the expression of circLONP2 and miR-584-5p and investigate their functions on cell proliferation and invasion. Luciferase reporter assay and RNA pull-down were performed to confirm the functional interactions among circLONP2/miR-584-5p/YAP1. CircLONP2 was significantly upregulated in precancerous BC tissues and BC cells. CircLONP2 depletion inhibited cell viability, proliferation, and invasion of BC cell lines, which could be partially rescued by miR-584-5p inhibitor. Further experiments indicated that miR-584-5p regulates cell viability, proliferation, and invasion via directly targeting YAP1. In summary, our work indicates that circLONP2 plays an oncogenic function in BC by regulating miR-584-5p/YAP1 axis, and its interaction with miR-584-5p provides a potential strategy to target BC.

## Graphical abstract

**Figure:**
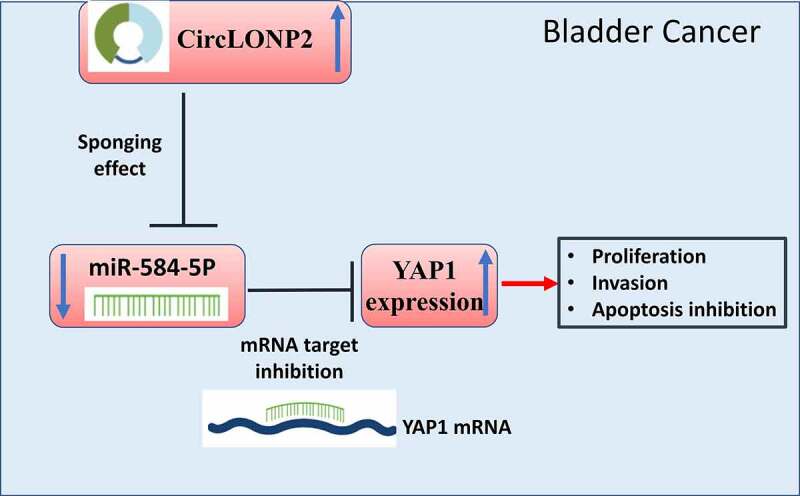

## Introduction

Bladder cancer (BC) is one of the most common malignancies in women and has the fourth highest incidence rate in men [1], which poses a serious threat to public health. BC can be further classified as different subtypes, such as non-muscle-invasive BC, muscle-invasive BC or as a metastatic form [2]. Among them, metastasis happens more frequently in muscle-invasive BC patients and usually correlates with poor prognosis [3,4]. The current treatments for diagnosed BC patients include an extended lymph node dissection, chemotherapies in neoadjuvant and adjuvant settings, and immunotherapy with bacillus Calmette–Guerin; however, 5‐year survival rate of metastatic BC is dismally low [5]. Therefore, understanding the underlying mechanisms of the invasion and metastasis of BC cells could provide valuable insights into the formulation of novel therapeutic intervention.

Circular RNAs (circRNAs) are a newly discovered class of non-coding RNAs that are closed-loop RNAs produced from back-splicing of precursor mRNAs [6,7]. Emerging data have revealed that circRNAs are associated with cellular differentiation and tissue homeostasis by targeting different downstream genes via post-transcriptional regulation [8,9]. For instance, circRNAs can modulate linear RNA transcription [10], act as microRNA (miRNA) sponges [11], interact with RNA-binding proteins (RBPs) [12], and are occasionally translated into small peptides [13]. In particular, recent studies have implicated circRNAs in tumor growth, metastasis, and drug resistance of BC [14]. There is evidence that circLONP2 is highly expressed in colorectal cancer and plays an oncogenic role [15]. However, the expression level and biological role of circLONP2 in BC remain to be investigated.

MicroRNAs (miRNAs) are a class of endogenous non-coding RNAs with 18–22 nucleotides in length, which are capable of regulating translation or modulating degradation of target mRNAs via binding to the 3’-untranslated regions (3’-UTR) [16]. By binding to microRNAs, circRNAs often serves as a potent inhibitor of miRNA activity [7]. The deregulation of microRNAs is widely reported in multiple cancers, including BC [^17–19^]. A number of studies have shown that miR-584-5p is down-regulated in a variety of tumors and seems to function as a tumor suppressor [^20–22^]. Nevertheless, currently there is no reported role of miR-584-5p in BC. YAP1 (Yes1 Associated Transcriptional Regulator) is a major downstream effector of the Hippo pathway, which serves as a transcriptional regulator in cell proliferation and survival. Its nuclear localization is closely linked with the development and proliferation of neoplasia [^23–25^]. Therefore, understanding the regulation of YAP1 expression in BC could provide insights into the development of novel targeted therapy.

In this study, we first showed that circLONP2 was significantly upregulated in precancerous BC tissues and BC cells. Since circLONP2 could interact with miR-584-5p based on the prediction by online bioinformatics tool, we hypothesized that circLONP2/miR-584-5p axis may regulate the malignant phenotype pf BC cells. We performed molecular experiments to validate the functional role of circLONP2 in BC, as well as its interplay with miR-584-5p. We further showed that miR-584-5p could interact with YAP1 and negatively regulated its expression. Together, our work suggests that circLONP2 acts as an oncogenic factor by targeting miR-584-5p/YAP1 axis, and its interaction with miR-584-5p provides a potential strategy for BC treatment.

## Methods

### Cell culture and treatment

BC cell lines (RT4, T24, EJ, UMUC3, 5637) and uroepithelium cell lines (SV-HUC-1) were obtained from the China Infrastructure of Cell Line Resource. All the cell lines were cultured in Dulbecco’s-modified Eagle’s medium (DMEM) (HyClone, USA) containing 10% FBS (FBS, Gibco, USA) and 100 IU/ml penicillin (GIBCO, UK) at 37°C in a humidified incubator.

### Cell proliferation assay

Cell proliferation was evaluated with CCK-8 assay kit in 96-well culture plates as previously described [26]. 48 hours after transfection, cells were seeded in to a 96-well plate at a density of 2000 cell per well, and cultured for 0, 24, 48, 72 and 96 h. 10 μL CCK8 reaction solution (Solarbio, Shanghai, China) was added to the cell culture at indicated time point and the cells were incubated for 1 h in a cell incubator. The light absorption value in each condition was captured at 450 nm wavelength on a microplate reader.

### Cell transfection

Small interfering RNA (siRNA), and shRNA of circLONP2 and the negative control si-NC and sh-NC were purchased from Hanbio (Hangzhou, China). Transfection in T24 and 5637 cells was performed with Lipofectamine 2000 (Invitrogen) based on manufacturer’s instruction. 50 nM of each molecule was used for transfection in 6 well plate with 60–70% confluency, and functional experiments were performed 48 hours post-transfection.

### Apoptosis

The detection of apoptosis was assessed using FITC Annexin V Apoptosis Detection Kit (Biolegend, California, USA), as previously described [27]. T24 and 5637 cells after different treatment were resuspended in Annexin V binding buffer at a concentration of 1.0 × 10^7^ cells/mL. Subsequently, 5 μL Annexin V-FITC and 5 μL PI solution were added to the cell resuspension and the cells were incubated for 30 mins in the dark. Stained cells were centrifuged and washed twice with Annexin V binding buffer and resuspended in 400 μL Annexin V binding buffer. The percentage of apoptotic events was analyzed by BD FACS CantoTM II Flow Cytometer (BD Biosciences).

### Edu incorporation assay

Edu incorporation assay was performed as previously described [28]. Click-iT™ EdU Cell Proliferation Kit for Imaging, Alexa Fluor™ 555 (Thermo Fisher Scietufic, USA) was used to detect cell proliferation. 1xEdU solution was added in the cell culture medium, and the cells were incubated for 2 h. The medium was discarded and cells were fixed with 100 µL of 3.7% formaldehyde in PBS for 15 min at room temperature. After the removal of fixative solution, cells were washed twice with PBS and permeabilized with 100 µL of 0.5% Triton® X-100 in PBS for 20 min. Then 1 x Click-iT® reaction cocktail was added to premetallized cells for 30-min incubation. The staining cocktail was removed and cells was washed twice with PBS. Cells were counter-stained with 500 nM DAPI in PBS and the images were captured under Leica AM6000 microscope (Leica, Wetzlar, Germany).

### Transwell migration and invasion assay

Transwell assay was performed as previously described [29]. For migration and invasion assay, we used transwell chambers without or with Matrigel (Corning, New York, Madison, USA). Cells were prepared at a density of 2 × 10^6^ cells/mL, and then 400 μL of cell suspension was inoculated into the upper chamber in serum-free medium, while 10% FBS-DMEM was added to the lower chamber. After incubation at 37°C for 24 h, cells were fixed with 10% methanol (Biotechnology, Shanghai, CN) for 10 min and stained with 0.5% crystal violet (Solarbio Co., Ltd, Beijing, CN) for 20 min at room temperature. Then, the stained cells were counted under a light microscope (Olympus, Tokyo, Japan).

### Luciferase reporter assays

Luciferase reporter assay was performed according to previously described [29]. PmirGLO dual-luciferase reproters (Promega, Madison, WI, USA) containing WT binding sequence for miR-584-5p or mutated sequence were co-transfected with miR-584-5p mimic or miR-NC at 100 nM using Lipofectamine 2000 reagent. After 48 h, the relative luciferase activities were measured using Dual-Luciferase Reporter Assay Kit (Promega) on a Fluoroskan Ascent FL plate reader (Thermo Scientific, USA). The relative firefly luciferase activity in the reporter plasmid was normalized to that of Renilla luciferase.

### RNA pull-down

RNA pull-down experiment was performed as described in previous study [30]. Cell lysates were collected by IP lysis buffer (Beyotime, Shanghai, China) and were incubated biotinylated miR-584-5p and Control miR-NC. Ten percent of the lysates was reserved as the input. The mixture was further incubated with M-280 streptavidin magnetic beads (Sigma-Aldrich, Germany) at 4°C for 4 h. A magnetic bar was used to pull down the magnetic beads and associated nucleic acids. The beads were washed 4 times with lysis buffer, and the total RNA in the input and the pull-down samples were purified with Trizol reagent (Invitrogen, Carlsbad, CA, USA) according to the manufacturer’s protocol. qRT-PCR was performed to quantify the relative expression of circLONP2 in each sample.

## RNA FISH

RNAscope kit (Invitrogen, CA, United States) was used to perform fluorescence in situ hybridization (FISH) according to manufacturer’s instructions. Briefly, cells were fixed with 4% paraformaldehyde and permeabilized with 0.1% Triton X100. circLONP2 probe with Cy3 fluorescent dye (RiboBio Co. Ltd., Guangzhou, China) was applied at 50 nM for hybridization for 3 hours at 50° . Cells were placed on the slide in mounting media containing DAPI (Vector Lab, Inc., Burlingame, CA, United States). Images were observed under Leica AM6000 microscope (Leica, Wetzlar, Germany).

### Databases

Circinteractome was utilized to computationally predict the miRNA targets of circLONP2. Gene Expression Profiling Interactive Analysis (GEPIA: http://gepia.cancer-pku.cn/index.html) was used to analyze the level of circLONP2 in BC samples. The Starbase was used to analyze the binding sequence between miR-584-5p and YAP1 mRNA.

### Western blot analysis

Western blot was conducted as described in our previous study with modifications [31]. 10–30 ug of total protein samples were loaded to 12% SDS-PAGE gel for electrophoresis. Separated protein in SDS-PAGE gel was transferred onto the PVDF membrane and the membrane was blocked with 5% skimmed milk for 1 h. The membrane was then incubated with primary antibodies: anti-YAP1 (1:1000, Affinity, CN) and anti-β-actin (1:5000, GeneTex, USA). The membrane was washed 3 times with TBST buffer and further incubated with HRP-linked secondary antibody (1:3000; Cell Signaling Technologies, MA, USA) at room temperature for 1 h. The protein bands were visualized using an enhanced chemiluminescence kit (Santa Cruz, TX, USA,) and photographed on a gel imager system (Bio-Rad, Hercules, CA, USA).

### Patients and tissue samples

Primary BC tumor samples and para-cancerous normal tissues were collected from surgical waste from patients diagnosed with BC at The Affiliated Nanping First Hospital of Fujian Medical University. The clinical parameters of the patients are summarized in Table 1. The study was approved by the Institutional Ethics Committee of the Affiliated Nanping First Hospital of Fujian Medical University. All patients signed informed consent.

**Table 1.** Correlation of circLONP2 with clinical features of BC patients

Chinicopathological characteristics	Total	high expression	low expression	X2	P value
Gender					
male	26	16	10	0.156	0.693
female	34	19	15		
Age					
≤60	33	20	13	0.194	0.660
>60	27	15	12		
Tumor size					
T1	12	2	10	17.196	0.001
T2	15	7	8		
T3	14	9	5		
T4	19	17	2		
Distant metastasis					
Positive	35	28	9	11.944	0.001
Negtive	25	7	16		
Differentiation					
high	25	20	5	13.413	0.001
moderate	23	13	10		
poor	12	2	10		
Lymph node metastasis					
Positive	29	25	7	11.051	0.001
Negtive	31	10	18		
TNM stages					
I	14	2	12	18.104	0.000
II	13	7	6		
III	12	8	4		
IV	21	18	3		

### Quantitative RT-PCR

According to our previous study [31], total RNA was extracted by TRIzol Reagent (Invitrogen, Carlsbad, CA, USA) as per the instruction. 1 µg of total RNA was converted into cDNA using the PrimeScript RT kit (Takara, Tokyo, Japan). qPCR was conducted to analyze gene expression using SYBR Green® Premix Ex Taq (Takara) using a 7500 Real-Time PCR System (Applied Biosystems, Carlsbad, CA, USA). The PCR cycling condition used: 95°C 2 mins, 40 cycles of 95°C 30 sec, 60°C 30 sec and 72°C 60 sec. 2–∆∆Ct method was used to analyze the relative expression level and GAPDH was used as the internal reference gene. For nucleoplasm fraction experiment, the nuclear and cytoplasmic faction was extracted using NE-PER™ Nuclear and Cytoplasmic Extraction Reagents (Thermo Fisher Scientific, Carlsbad, CA, USA). The primer sequences were as follows (from 5’ – 3’): circLONP2 forward primer: GACTGAGAGAGAAGGCGCAC, reverse primer: TGGGTTGTTCACTCCCACAG; miR-584-5p forward primer: TTATGGTTTGCCTGGGACTGAG, reverse primer: GCGAGCACAGAATTAATACGAC; GAPDH froward primer: TGCACCACCAACTGCTTAGC, reverse primer: GGCATGGACTGTGGTCATGAG; and U6 froward primer: CTCGCTTCGGCAGCACA, reverse primer: AACGCTTCACGAATTTGCGT.

### Xenograft tumor in nude mice

All animal care and use procedures were approved by the Institutional Ethics Committee of the Affiliated Nanping First Hospital of Fujian Medical University. Twelve male immunodeficient nude mice (12-weekold) were randomly divided into two groups (six mice in each group): (1) si-NC group (injected with T24 cells transected with sh-NC), (2) sh‐circLONP2 (injected with T24 cells infected with sh‐circLONP2 knockdown). 0.25 mL of cell suspension containing 0.5 × 10^7^ cells was injected into the flank of each mice. Tumor volume were monitored for 4 weeks. At the end of the experiment, all the mice were euthanized by CO_2_ asphyxiation. The tumors of terminally dead mice were resected for weight measurement and fixed for further analysis.

### Immunohistochemistry

Histological analyses of tumor sections were performed with the avidin-biotin-peroxidase method to detect ki-67 protein as previously described [32]. Briefly, the tumor tissues were cut into 4-μm-thick paraffin sections, then they were deparaffinized and rehydrated, and antigen unmasking was achieved by heating the section in citrate unmasking solution (SignalStain® Citrate Unmasking Solution (10X), Cell Signaling Technologies) for 10 min at a sub-boiling temperature (95°–98°C). Sections were washed in dH2O three times and then incubated in 3% hydrogen peroxide for 10 min. After three times washes in TBST buffer, the section was blocked with 5% Normal Goat Serum, and stained with primary antibody anti-Ki67 (1:500 dilution, Cell Signaling Technologies) overnight at 4°C. Then antibody solution was removed and the sections were washed with TBST buffer three times. The sections were further soaked in 1–3 drops of SignalStain® Boost Detection Reagent (HRP, Rabbit, Cell Signaling Technologies) for 30 min at room temperature. 300 µl SignalStain® substrate (Cell Signaling Technologies) was added to each section for 5 min. After washes in dH2O the sections were mounted with coverslips using the mounting medium (Cell Signaling Technologies) and imaged under Leica AM6000 microscope (Leica, Wetzlar, Germany).

### Statistical analysis

Statistical analyses were performed with GraphPad Prism software, version 7.0 (GraphPad Software). Differences between percentages were evaluated by Fisher’s exact test. The statistical difference between two groups was examined by unpaired Student’s *t* test. Comparisons among multiple groups were analyzed using one-way analysis of variance (ANOVA) with Tukey’s post hoc test. Kaplan Meier Curve and log-rank test were used to compare the cumulative survival rates in BC patients. *P* < 0.05 was considered statistically significant.

### Results

This study investigated the role of circLONP2 in BC. We showed that circLONP2 was significantly upregulated in precancerous BC tissues and BC cells. Since circLONP2 is predicted to interact with miR-584-5p using the online bioinformatics tool, we performed molecular experiments to validate the functional role of circLONP2 in BC, as well as its interplay with miR-584-5p. In addition, miR-584-5p could target YAP1 mRNA and negatively regulated its expression. Together, our work suggests that circLONP2 acts as an oncogenic factor by targeting miR-584-5p/YAP1 axis, and its interaction with miR-584-5p provides a potential strategy for BC treatment.

### CircLONP2 is upregulated in BC

**Figure 1.**
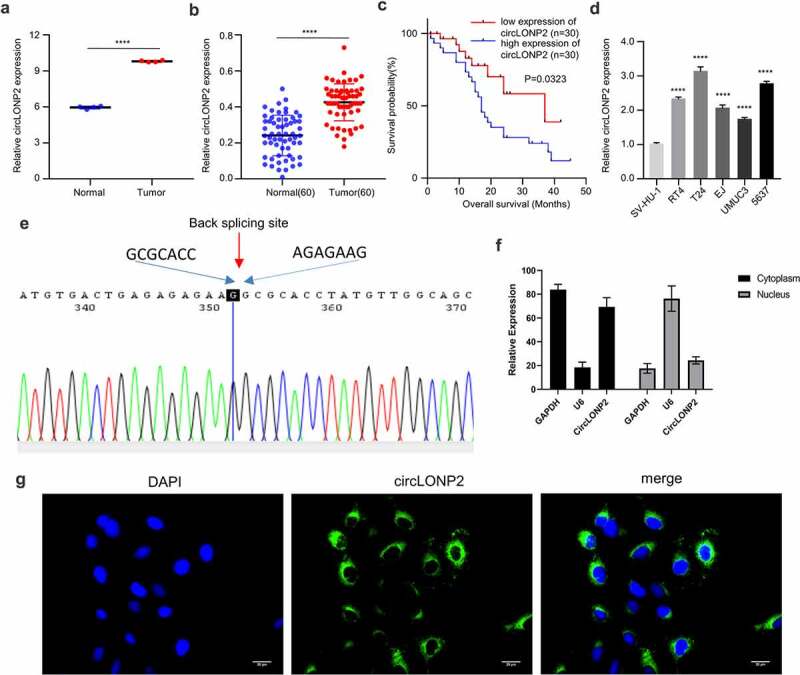
CircLONP2 is highly expressed in BC tissues and cells. (a). The analysis of GSE92675 RNA-seq dataset for CircLONP2 expression. (b). The expression level of circLONP2 in 60 pairs of BC tumors and para-cancerous normal tissues was examined by qRT-PCR. (c). Kaplan–Meier survival assessment of BC patients, which were divided into low and high circLONP2 expression groups (n = 60). (d). The expression level of circLONP2 in BC cell lines (RT4, T24, EJ, UMUC3 and 5637) and normal bladder cell line (SV-HU-1) (n = 3). (e). Sanger sequencing showing the specific back splicing site of circLONP2. (f). The relative expression level of GAPDH, U6 and circLONP2 in the cytoplasmic and nuclear faction of T24 cells (n = 3). G. RNA FISH showing the predominant cytoplasmic loclaization of circLONP2 in T24 cells. *P < 0.05 versus Control, **P < 0.01, ****P < 0.0001.

We first retrieved RNA-seq data from Gene Expression Omnibus (GSE92675) to investigate the expression of circLONP2 between BC tissues and para-cancerous normal tissues. CircLONP2 was highly expressed in BC tissues as compared to normal tissues (Figure 1(a)). In addition, we collected 60 paired BC tumors and para-cancerous normal tissues samples from 60 BC patients. qRT-PCR analysis showed that level of circLONP2 in para-cancerous tissue samples were significantly higher than that of normal tissues (Figure 1(b)). Based on the median expression value of circLONP2, 60 BC patients were divided into circLONP2 low and high-expression groups (n = 30 in each group), and chi-square test was used to analyze the association of circLONP2 expression level and clinicopathological features. The results indicate that circLONP2 expression is positively correlated with tumor size, differentiation status, TNM staging, lymph node metastasis and distant metastasis, while it shows no significantly correlation with the age and gender of patients (Table 1). The overall survival rate (OS) of patients analyzed by Kaplan–Meier curve demonstrated that high circLONP2 expression is significantly correlated with a poorer prognosis in BC patients (Figure 1(c)). In addition, we analyzed the expression of circLONP2 in BC cell lines (RT4, T24, and EJ, UMUC3, 5637) and normal bladder epithelial cell line (SV-HU-1), which revealed a significant upregulation of circLONP2 in BC cell lines (Figure 1(d)). These data indicate that circLONP2 upregulation may contribute to the progression of BC.

To confirm the circular structure of circLONP2, we performed Sanger sequencing to examine the sequence in the closed-loop structure, which verified the specific back-splicing site and the existence of circLONP2 (Figure 1(e)). We next performed nuclear and cytoplasmic fraction to analyze the relative distribution of circLONP2. The results showed circLONP2 is predominantly located in the cytoplasm (Figure 1(f)). This was further confirmed by RNA fluorescence in situ hybridization (FISH) (Figure 1(g)).

### The knockdown of circLONP2 inhibits tumor cell proliferation, migration, and invasion

**Figure 2.**
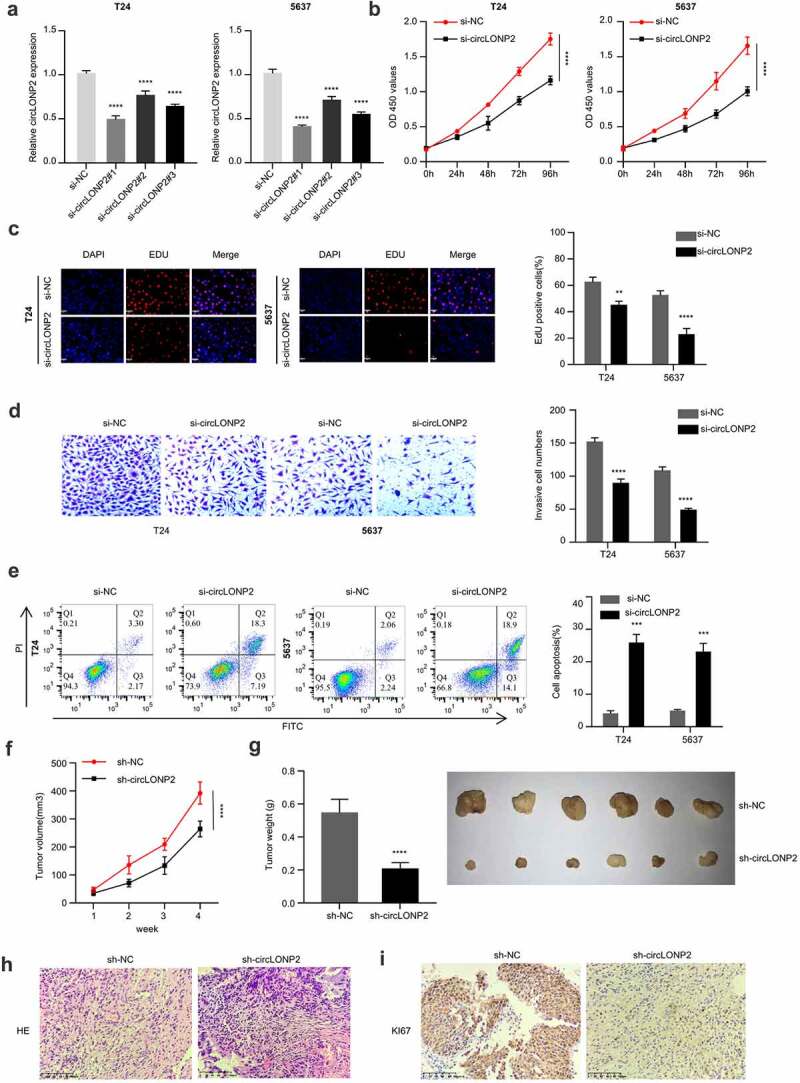
Knockdown of circLONP2 inhibits the proliferation and invasion and induced apoptosis of BC cells. (a). The efficiency of circLONP2 knockdown was verified by qRT-PCR. (b). CCK-8 proliferation assay in T24 and 5637 cells after transfecting with si-NC and si-circLONP2 siRNA (n = 6). (c). Edu incorporation assays in in T24 and 5637 cells after transfecting with si-NC and si-circLONP2 siRNA (n = 3). (d). Transwell invasion assay, and (e). apoptosis analysis in T24 and 5637 cells transfected with si-NC and si-circLONP2 (n = 3). (f-g). Tumor growth curve and subcutaneous tumor weight of mice inoculated with T24 cells transfected with sh-NC and sh-circLONP2 (n = 6 mice in each group). (h-i). The representative H&E staining and Ki67 immunohistochemical staining images of the subcutaneous tumors in sh-NC and sh-circLONP2 group. *P < 0.05 versus Control, **P < 0.01, ****P < 0.0001.

We selected the two BC cell lines with the highest expression of circLONP2 (T24 and 5637) and applied siRNAs to investigate the effect of circLONP2 knockdown in BC cells. Compared with si-NC, si-RNA transfection (si-circLONP2#1, si-circLONP2#2 and si-circLONP2#3) effectively reduced circLONP2 level in the two cell lines. Among them, si-circLONP2^#1^ showed the highest knockdown efficiency, which is applied for the following experiments (Figure 2(a)). CCK-8 proliferation assay demonstrated a significant impairment in cell proliferation after silencing circLONP2 (Figure 2(b)). This was accompanied by the reduced incorporation of EdU into DNA, indicating retarded DNA synthesis (Figure 2(c)). Transwell invasion assay also revealed the impaired invasion ability after silencing circLONP2 (Figure 2(d)). Silencing of circLONP2 also caused increased apoptotic events in T24 and 5637 cell lines (Figure 2(e)). Together, these data suggest circLONP2 is required for the malignant phenotype of BC cells.

To further investigate the role of circLONP2 in tumorigenesis, T24 cells transfected with si-NC or si-circLONP2 were subcutaneously injected into 12 nude mice (n = 6 in each group). The silencing of circLONP2 significantly reduced tumorigenesis in nude mice, as revealed by the decreased tumor volume and weight (Figure 2(f–g)). We also examined the level of ki-67 expression in tumor section via immunohistochemistry. In the sh-circLONP2 group, there was a remarkable decrease of Ki-67 signal in the tumor section (Figure 2(h–j)). Overall, these data indicate that circLONP2 is indispensable for tumorigenesis in nude mice.

### MiR-584-5p expression is downregulated by circLONP2 in BC cell lines

**Figure 3.**
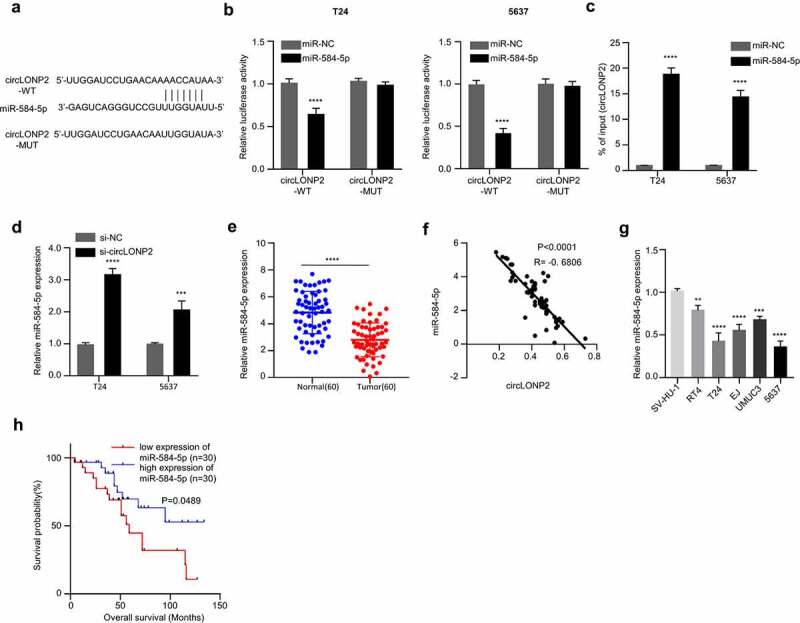
CircLONP2 targets miR-584-5p. (a). Alignment of the miR-584-5p sequence with that of circLONP2, showing their binding site. (b). Dual-luciferase reporter assay using WT and MUT reporter to determine the interplay between circLONP2 and miR-584-5p in T24 and 5637 cells. Cells were co-transfected with miR-NC or miR-548-5p mimic. (n = 3) (c). Verification of physical interaction between circLONP2 and miR-584-5p through RNA pull-down using biotin-labeled miR-548-5p probe. (n = 3). (d) The level of miR-584-5p in T24 and 5637 cells transfected with si- circLONP2. (n = 3). (e). The level of miR-584-5p in 60 BC tumors and para-cancerous normal tissues by qRT-PCR. (f). Spearman’s correlation analysis of the expression levels between circLONP2 and miR-584-5p in 60 BC tissues. G. The expression of miR-584-5p in BC cell lines (RT4, T24, EJ, UMUC3 and 5637) and normal bladder epithelial cell line (SV-HU-1) by qRT-PCR. (h). Kaplan–Meier survival assessment of 60 BC patients, which were divided into miR-584-5p low and high expression groups (n = 30 in each group, P = 0.0489). **P < 0.01, ***P < 0.001, ****P < 0.0001.

We next sought to find the downstream target of circLONP2. Based on the predictive analysis of the bioinformatics database (circinteractome), we found that circLONP2 has a binding site for miR-584-5p (Figure 3(a)). We then performed dual-luciferase reporter assay using the reporter containing WT and mutated binding site. The co-transfection of miR-584-5p mimic could significantly reduce the luciferase activity of circLONP2 WT reporter, while it had no effect on the mutated reporter (Figure 3(b)). Furthermore, RNA pull-down assay suggested that biotin-labeled miR-584-5p probe could sufficiently enrich circLONP2 in T24 and 5637 cells (Figure 3(c)). These results suggest that circLONP2 physically interacts with miR-584-5p.

Next, we assessed expression of miR-584-5p in T24 and 5637 cells after silencing circLONP2. The transfection of si-circLONP2 significantly upregulated miR-584-5p (Figure 3(d)). In the 60 pairs of BC tumors and para-cancerous normal tissues, there was a significant downregulation of miR-584-5p (Figure 3(e)), and the expression of miR-584-5p was negatively correlated with circLONP2 level in BC tumors (Figure 3(f)). The downregulation of miR-584-5p was also detected in BC cell lines (RT4, T24, EJ, UMUC3 and 5637) (Figure 3(g)). Kaplan–Meier curve indicated a better overall survival in BC patients with high miR-584-5p expression (Figure 3(h)). Altogether, these data indicate circLONP2 negatively regulates miR-584-5p expression in BC cells.

### MiR-584-5p targets YAP1 and negatively regulates its expression

**Figure 4.**
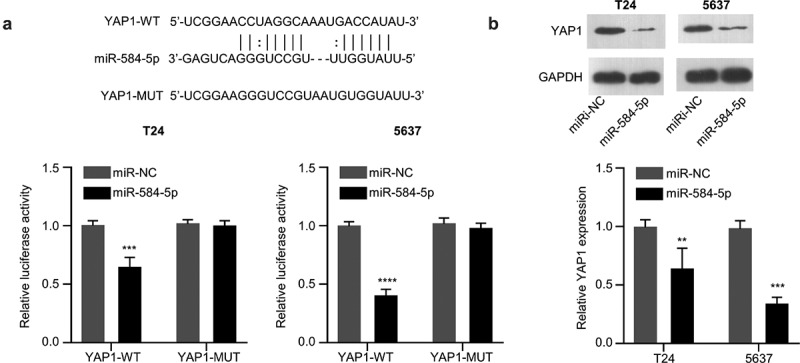
MiR-584-5p targets YAP1. (a). Alignment of the miR-584-5p sequence with that of the 3’-UTR of YAP1 mRNA, which was determined by Dual-luciferase reporter assay in T24 and 5637 cells. (n = 3). (b). Representative western blot and densitometric analysis of YAP1 protein level in miR-584-5p-overwxpressed T24 and 5637 cells (n = 3). **P < 0.01, ***P < 0.001, ****P < 0.0001.

We next found that miR-584-5p has a binding site in the 3’UTR region of YAP1 mRNA through Starbase online bioinformatic tool. We performed dual-luciferase activity assay and demonstrated that the transfection of miR-584-5p mimic could significantly impair the activity of WT reporter, while no effect was observed for the mutated reporter (Figure 4(a)). These results indicate the functional interaction between miR-584-5p and YAP1 mRNA. In addition, in the presence of miR-584-5p mimic, the protein expression level of YAP1 in T24 and 5637 cells was significantly decreased (Figure 4(b)). Therefore, miR-584-5pfunctiosn as negative regulator of YAP1.

### CircLONP2 regulates the progression of BC through the miR-584-5p/YAP1 axis

**Figure 5.**
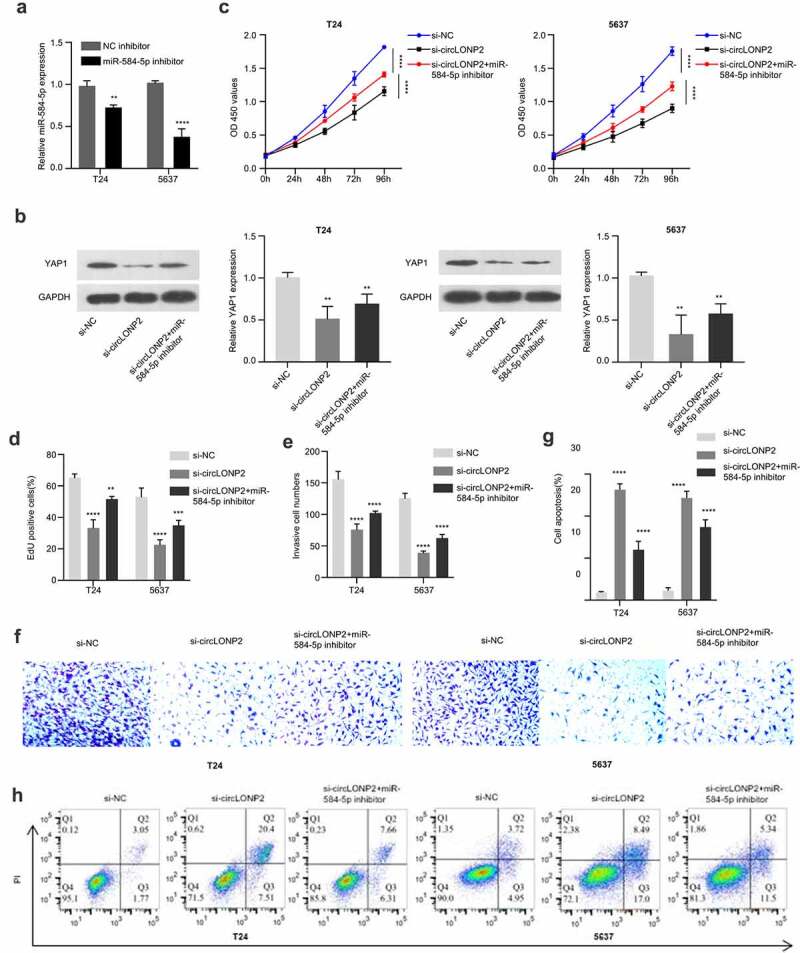
CircLONP2 regulates the progression of BC cells through the miR-584-5p/YAP1 axis. (a). Expression level of miR-584-5p in T24 and 5637 cells transfected NC inhibitor or miR-584-5p inhibitor (n = 3). (b). Representative Western blot and densitometric analysis of YAP1 in T24 and 5637 cells transfected with si-NC, si-circLONP2 and si-circLONP2+ miR-584-5p inhibitor (n = 3). (c). CCK-8 proliferation assay in T24 and 5637 cells after transfected with si-NC, si-circLONP2 and si-circLONP2+ miR-584-5p inhibitor (n = 6). (d). Edu incorporation assay, (e-f). Transwell invasion assay and (g-h). apoptosis analysis in T24 and 5637 cells transfected with si-NC, si-circLONP2 and si-circLONP2+ miR-584-5p inhibitor (n = 3). **P < 0.01, ***P < 0.001, ****P < 0.0001.

In order to explore the role of circLONP2/miR-584-5p/YAP1 axis n the regulation of malignant phenotype of BC cells, we transfected an inhibitor of miR-584-5p into T24 and 5637 cells, which could significantly lower the expression level of miR-584-5p (Figure 5(a)). Next, we divided T24 and 5637 cells into three groups, si-NC, si-circLONP2 and si-circLONP2+ miR-584-5p inhibitor, the expression of YAP1 was evaluated by Western blot. As expected, YAP1 level was significantly reduced after circLONP2 knockdown, which could be partially rescued by miR-584-5p inhibitor (Figure 5(b)). CCK-8 proliferation assay revealed that the suppression of cell proliferation by circLONP2 knockdown was partially relieved with miR-584-5p inhibitor (Figure 5(c)). A similar effect was observed in EdU incorporation assay (Figure 5(d)). The presence of miR-584-5p inhibitor also mitigated the inhibition of cell invasion caused by circLONP2 knockdown, and decreased apoptotic events in T24 and 5637 cells (Figure 5(e–h)).

## Discussion

In the era of precision medicine, targeted therapy for specific molecules has become an promising direction of drug development and oncology clinical care [33]. While there were limited advances in clinical management of BC over the past three decades, this trend has been considerably altered over the past several years. Accumulating clinical data indicate that BC ranks the highest among urinary tumors worldwide, with a poor prognosis and high recurrence rate [34]. The complex etiology and pathophysiology of BC makes it difficult to devise precise medicine.

For the past 7 years, caner genomic profiling and checkpoint blockade immunotherapy have significantly revolutionized cancer therapy [2]. The identification of the genes dysregulated in BC is of great value for targeted therapy. CircLONP2 is an endogenous circular non-coding RNA, which has been reported to be highly expressed in colorectal cancer and plays an oncogenic role [15]. Compared with linear RNA molecules, circRNAs are a type of highly abundant single-stranded non-coding RNAs in mammalian cells [35,36]. Owing to its circular structure and stability, circRNAs can function as miRNA sponges, regulate transcription or translation, and also form circRNA-protein interactions [35,36]. The aberrant expression of circRNAs could serve as diagnostic and prognostic biomarkers [37]. In the present study, we demonstrated the upregulation of circLONP2 in BC tissues and cells, and a high circLONP2 expression level is positively closed to tumor size, differentiation status, TNM classification, lymph node metastasis, and distant metastasis. Importantly, the knockdown of circLONP2 suppresses the proliferation and invasion of BC cells, and induces apoptosis in BC cells. Additionally, the overall survival of BC patients in circLONP2-high patients worse, indicating that circLONP2 may serve as a prognostic indicator in BC patients.

One of the dominant roles of circRNAs is to function as miRNA sponges [38,39]. miRNAs can act as either oncogenes or tumor-suppressors via interacting with target mRNAs. In cancer biology, miRNAs are implicated in a variety of malignancies by modulating different downstream targets [^40–43^]. In BC cells, miRNAs are involved in the regulation of cell proliferation, migration, and invasion, self-renewal, angiogenesis, as well as the development of resistance [^44–48^]. In our results, we further revealed that circLONP2 could bind to miR-584-5p and inhibit its function. Since there are no reports regarding the role of miR-584-5p in BC, our study provided novel evidence of miR-584-5p in the regulation of malignant phenotype of BC. CircLONP2 can physically interact with miR-584-5p and the level of miR-584-5p is negatively regulated by circLONP2. Moreover, the expression of miR-584-5p in BC tumor tissues and BC cell lines is significantly lower than that in normal tissues and cell lines. All these evidence supports the tumor-suppressor role of miR-584-5p in BC.

We further showed that miR-584-5p could bind to the 3’UTR region of YAP1 mRNA to suppress YAP1 expression. Overexpression of miR-584-5p effectively reduces the expression of YAP1 in T24 and 5637 cells. YAP1 is a major regulator of the Hippo signaling pathway, which is frequently upregulated in BC cells to sustain cell proliferation, invasion, and apoptosis resistance [25,49,50]. YAP1 expression has also been proposed as a prognostic indicator in patients with urothelial carcinoma in the bladder [51]. Therefore, specific inhibitors for YAP1 activity are an attractive approach to improve treatment for BC. In addition, we also observed that the knockdown of circLONP2 reduces the protein level of YAP1, while co-transfection of miR-584-5p inhibitor can partially increase YAP1 level. Meanwhile, the effect of circLONP2 silencing in cell proliferation and invasion could also be partially alleviated by miR-584-5p inhibitor. These data altogether provide evidence for the modulation of circLONP2/miR-584-5p/YAP1 axis in the management of BC progression.

## Conclusion

Limited advancement has been achieved in the understanding of molecular mechanisms underlying the initiation and progression of BC [^52–55^]. Our study adds novel evidence for the oncogenic role of circLONP2 in supporting the malignant phenotype of BC, as well as its value as a prognostic indicator in BC patients. We further identified miR-584-5p as a downstream mediator of circLONP2, which interacts with YAP1 mRNA to negatively regulate its expression. These results altogether indicate that modulating circLONP2/miR-584-5p/YAP1 axis may serve as a potential strategy in the management of BC progression.

## Data Availability

The data is available from the corresponding author on reasonable request.

## References

[1] Lenis A, Lec P, Chamie K, et al. Bladder Cancer: a Review. JAMA. 2020;324(19):1980–1991. 33201207 10.1001/jama.2020.17598

[2] Tran L, Xiao JF, Agarwal N, et al. Advances in bladder cancer biology and therapy. Nat Rev Cancer. 2020;21:104–121. 33268841 10.1038/s41568-020-00313-1 PMC10112195

[3] Kamat AM, Hahn NM, Efstathiou JA, et al. Bladder cancer. Lancet. 2016;388(10061):2796. 27345655 10.1016/S0140-6736(16)30512-8

[4] Witjes JA, Lebret T, Compérat E, et al. Updated 2016 EAU guidelines on muscle-invasive and metastatic bladder cancer. Eur Urol. 2016;71:(3)–462–475. 27375033 10.1016/j.eururo.2016.06.020

[5] Abdollah F, Gandaglia G, Thuret R, et al. Incidence, survival and mortality rates of stage-specific bladder cancer in United States: a trend analysis. Cancer Epidemiol. 2013;37(3):219–225. 23485480 10.1016/j.canep.2013.02.002

[6] Kristensen LS, Hansen TB, Ven MT, et al. Circular RNAs in cancer: opportunities and challenges in the field. Oncogene. 2018;37(5):555–565. 28991235 10.1038/onc.2017.361 PMC5799710

[7] Kristensen LS, Andersen MS, Stagsted L, et al. The biogenesis, biology and characterization of circular RNAs. Nat Rev Genet. 2019;20(7):675–691. 31395983 10.1038/s41576-019-0158-7

[8] Salzman J. Circular RNA expression: its potential regulation and function. Trends Genet. 2016;32(5):309–316. 27050930 10.1016/j.tig.2016.03.002 PMC4948998

[9] Hansen TB, Venø M, Damgaard CK, et al. Comparison of circular RNA prediction tools. Nucleic Acids Res. 2016;6:e58–e58. 10.1093/nar/gkv1458 PMC4824091 26657634

[10] Zhaoyong L, Chuan H, Chun B, et al. Exon-intron circular RNAs regulate transcription in the nucleus. Nat Struct Mol Biol. 2015;22(3): 256–264. 25664725 10.1038/nsmb.2959

[11] Hansen TB, Jensen TI, Clausen BH, et al. Natural RNA circles function as efficient microRNA sponges. Nature. 2013;495(7441):384–388. 23446346 10.1038/nature11993

[12] Conn S, Pillman K, Toubia J, et al. The RNA binding protein quaking regulates formation of circRNAs. Cell. 2015;160(6):1125–1134. 2018. 25768908 10.1016/j.cell.2015.02.014

[13] Legnini I, Timoteo GD, Rossi F, et al. Circ-ZNF609 is a circular RNA that can be translated and functions in myogenesis. Elsevier Sponsored Documents. 2017;66(1):22–37.e9. 10.1016/j.molcel.2017.02.017 PMC5387670 28344082

[14] Yang X, Ye T, Liu H, et al. Expression profiles, biological functions and clinical significance of circRNAs in bladder cancer. Mol Cancer. 2021;20(1). DOI:10.1186/s12943-020-01300-8 PMC7780637 33397425

[15] Han K, Wang F, Cao C, et al. CircLONP2 enhances colorectal carcinoma invasion and metastasis through modulating the maturation and exosomal dissemination of microRNA-17. Mol Cancer. 2020;19(1):60. DOI:10.1186/s12943-020-01184-8 32188489 PMC7079398

[16] Bartel DP. MicroRNAs: genomics, biogenesis, mechanism, and function. Cell. 2004;116(2):281–297. 14744438 10.1016/s0092-8674(04)00045-5

[17] Hammouz R, Kołat D, Kałuzińska Ż, et al. MicroRNAs: their role in metastasis, angiogenesis, and the potential for biomarker utility in bladder carcinomas. Cancers (Basel). 2021;13(4):891. 33672684 10.3390/cancers13040891 PMC7924383

[18] Chen Y, Wang J, Wang D, et al. TNNT1, negatively regulated by miR-873, promotes the progression of colorectal cancer. J Gene Med. 2020;22(2):e3152. 31830337 10.1002/jgm.3152 PMC7027576

[19] Cheng Y, Zhang X, Li P, et al. MiR-200c promotes bladder cancer cell migration and invasion by directly targeting RECK. Onco Targets Ther. 2016;9:5091–5099. 27574450 10.2147/OTT.S101067 PMC4993393

[20] Xiang J, Wu Y, Li DS, et al. miR-584 suppresses invasion and cell migration of thyroid carcinoma by regulating the target oncogene ROCK1. Oncol Res Treat. 2015;38(9):436–440. 26405762 10.1159/000438967

[21] Lee SB, Park YS, Sung JS, et al. Tumor suppressor miR-584-5p inhibits migration and invasion in smoking related non-small cell lung cancer cells by targeting YKT6. Cancers (Basel). 2021;13(5):1159. 33800298 10.3390/cancers13051159 PMC7962648

[22] Ueno K, Hirata H, Shahryari V, et al. Tumour suppressor microRNA-584 directly targets oncogene Rock-1 and decreases invasion ability in human clear cell renal cell carcinoma. Br J Cancer. 2011;104(2):308–315. 21119662 10.1038/sj.bjc.6606028 PMC3031891

[23] Panciera T, Azzolin L, Cordenonsi M, et al. Mechanobiology of YAP and TAZ in physiology and disease. Nat Rev Mol Cell Biol. 2017;18(12):758–770. 28951564 10.1038/nrm.2017.87 PMC6192510

[24] Moroishi T, Hansen CG, Guan KL. The emerging roles of YAP and TAZ in cancer. Nat Rev Cancer. 2015;15(2):73–79. 25592648 10.1038/nrc3876 PMC4562315

[25] Ghasemi H, Mousavibahar SH, Hashemnia M, et al. Tissue stiffness contributes to YAP activation in bladder cancer patients undergoing transurethral resection. Ann N Y Acad Sci. 2020;1473(1):48–61. 32428277 10.1111/nyas.14358

[26] Miranda A, Blanco-Prieto M, Sousa J, et al. Breaching barriers in glioblastoma. Part I: molecular pathways and novel treatment approaches. Int J Pharm. 2017;531(1):372–388. 28755993 10.1016/j.ijpharm.2017.07.056

[27] Gao W, Jin Y, Hao J, et al. Hydroxyurea affects in vitro porcine oocyte maturation through increased apoptosis and oxidative stress. Biosci Rep. 2021;41(4). DOI:10.1042/bsr20203091 PMC8062957 33844009

[28] Serna-Salas S, Arroyave-Ospina J, Zhang M, et al. α-1 Adrenergic receptor antagonist doxazosin reverses hepatic stellate cells activation via induction of senescence. Mech Ageing Dev. 2021;201:111617. 34958827 10.1016/j.mad.2021.111617

[29] Guan B, Li G, Wan B, et al. RNA -binding protein RBM38 inhibits colorectal cancer progression by partly and competitively binding to PTEN 3’ UTR with miR-92a-3p. Environ Toxicol. 2021;36(12):2436–2447. 34453780 10.1002/tox.23356

[30] Luo L, Miao P, Ming Y, et al. Circ-ZFR promotes progression of bladder cancer by upregulating WNT5A via sponging miR-545 and miR-1270. Front Oncol. 2021;10(3310). DOI:10.3389/fonc.2020.596623 PMC8076638 33928018

[31] Tang W, Zhang L, Li Z. Long noncoding RNA LOC100911498 is a novel regulator of neuropathic pain in rats. Brain Behav. 2021;11(8):e01966. 33949153 10.1002/brb3.1966 PMC8413752

[32] Ma H, Jiang T, Tang W, et al. Transplantation of platelet-derived mitochondria alleviates cognitive impairment and mitochondrial dysfunction in db/db mice. Clin sci. 2020;134(16):2161–2175. 10.1042/CS20200530 32794577

[33] Velmahos C, Badgeley M, Lo Y. Using deep learning to identify bladder cancers with FGFR-activating mutations from histology images. Cancer Med. 2021;10(14):4805–4813. 34114376 10.1002/cam4.4044 PMC8290253

[34] Dobruch J, Daneshmand S, Fisch M, et al. Gender and bladder cancer: a collaborative review of etiology, biology, and outcomes. Eur Urol. 2016;69(2):300–310. 26346676 10.1016/j.eururo.2015.08.037

[35] Jeck WR, Sorrentino JA, Wang K. Circular RNAs are abundant, conserved, and associated with ALU repeats (2013). Rna. 2013;19(2):141–157. 23249747 10.1261/rna.035667.112 PMC3543092

[36] Wu S, Yang J, Xu H, et al. Circular RNA circGLIS3 promotes bladder cancer proliferation via the miR-1273f/SKP1/Cyclin D1 axis. Cell Biol Toxicol. 2021. DOI:10.1007/s10565-021-09591-3 PMC8789643 33656636

[37] Li A, Wang WC, Mcalister V, et al. Circular RNA in colorectal cancer. J Cell Mol Med. 2021;25(8):3667–3679. 33687140 10.1111/jcmm.16380 PMC8051750

[38] Cao W, Zhao Y, Wang L, et al. Circ0001429 regulates progression of bladder cancer through binding miR-205-5p and promoting VEGFA expression. Cancer Biomarkers. 2019;25(1):101–113. 30909190 10.3233/CBM-182380 PMC13082421

[39] Zhang Y, Wang H, Li C, et al. CircSMYD4 regulates proliferation, migration and apoptosis of hepatocellular carcinoma cells by sponging miR-584-5p. Cancer Cell Int. 2020;20(1):556. 33292243 10.1186/s12935-020-01648-3 PMC7678128

[40] Turai P, Nyírő G, Butz H, et al. MicroRNAs, long non-coding RNAs, and circular RNAs: potential biomarkers and therapeutic targets in Pheochromocytoma/Paraganglioma. Cancers (Basel). 2021;13(7):1522. 33810219 10.3390/cancers13071522 PMC8036642

[41] Lin Y, Chen T, Huang Y, et al. Involvement of microRNA in solid cancer: role and regulatory mechanisms. Biomedicines. 2021;9(4):343. 33805515 10.3390/biomedicines9040343 PMC8065716

[42] Liu Y, Li Y, Liu J, et al. Long noncoding RNA GAS5 targeting miR-221-3p/cyclin-dependent kinase inhibitor 2B axis regulates follicular thyroid carcinoma cell cycle and proliferation. Pathobiol J Immunopathol Mol Cell Biol. 2021;1–12. DOI:10.1159/000513338. 34130294

[43] Li Q, Li Z, Wei S, et al. Overexpression of miR-584-5p inhibits proliferation and induces apoptosis by targeting WW domain-containing E3 ubiquitin protein ligase 1 in gastric cancer. J Exp Clin Cancer Res. 2017;36(1):59. 28431583 10.1186/s13046-017-0532-2 PMC5401563

[44] Chen Y, Xu Y, Liu W, et al. Long noncoding RNA KCNMB2-AS1 promotes SMAD5 by targeting miR-3194-3p to induce bladder cancer progression. Front Oncol. 2021;11:649778. 34026626 10.3389/fonc.2021.649778 PMC8138055

[45] Monoe Y, Jingushi K, Kawase A, et al. Pharmacological inhibition of miR-130 family suppresses bladder tumor growth by targeting various oncogenic pathways via PTPN1. Int J Mol Sci. 2021;22(9):4751. 33947152 10.3390/ijms22094751 PMC8124864

[46] Lin J, Tsai K. Circulating miRNAs act as diagnostic biomarkers for bladder cancer in urine. Int J Mol Sci. 2021;22(8). DOI:10.3390/ijms22084278 PMC8074331 33924142

[47] Wang H, Niu X, Jiang H, et al. Long non-coding RNA DLX6-AS1 facilitates bladder cancer progression through modulating miR-195-5p/VEGFA signaling pathway. Aging (Albany NY). 2020;12(16):16021–16034. 32756011 10.18632/aging.103374 PMC7485696

[48] Yang X, Wang P. MiR-188-5p and MiR-141-3p influence prognosis of bladder cancer and promote bladder cancer synergistically. Pathol Res Pract. 2019;215(11):152598. 31562019 10.1016/j.prp.2019.152598

[49] Zhang J, Deng X. Effects of miR-599 targeting YAP1 on proliferation, invasion and apoptosis of bladder urothelial carcinoma cells. Exp Mol Pathol. 2021;118:104599. 33359177 10.1016/j.yexmp.2020.104599

[50] Li S, Zhu H, Chen H, et al. Glucose promotes epithelial-mesenchymal transitions in bladder cancer by regulating the functions of YAP1 and TAZ. J Cell Mol Med. 2020;24(18):10391–10401. 32678516 10.1111/jcmm.15653 PMC7521329

[51] Liu J, Li Y, Lin H, et al. Overexpression of YAP 1 contributes to progressive features and poor prognosis of human urothelial carcinoma of the bladder. BMC Cancer. 2013;13(1):349. 23870412 10.1186/1471-2407-13-349 PMC3750259

[52] Jiang L, Zuo Z, Lin J, et al. Orthodenticle homeobox OTX1 is a potential prognostic biomarker for bladder cancer. Bioengineered. 2021;12(1):6559–6571. 34559577 10.1080/21655979.2021.1974646 PMC8806575

[53] Huang R, Zheng Z, Xian S, et al. Identification of prognostic and bone metastatic alternative splicing signatures in bladder cancer. Bioengineered. 2021;12(1):5289–5304. 34402716 10.1080/21655979.2021.1964252 PMC8806927

[54] Yang J, Fan L, Liao X, et al. CRTAC1 (Cartilage acidic protein 1) inhibits cell proliferation, migration, invasion and epithelial-mesenchymal transition (EMT) process in bladder cancer by downregulating Yin Yang 1 (YY1) to inactivate the TGF-β pathway. Bioengineered. 2021;12(2):9377–9389. 34818994 10.1080/21655979.2021.1974645 PMC8809913

[55] Chen Y, Wang D, Shu T, et al. Circular RNA_0000326 promotes bladder cancer progression via microRNA-338-3p/ETS Proto-Oncogene 1/phosphoinositide-3 kinase/Akt pathway. Bioengineered. 2021;12(2):11410–11422. 34889689 10.1080/21655979.2021.2008738 PMC8810167

